# The SET protein promotes androgen production in testicular Leydig cells

**DOI:** 10.1111/andr.12476

**Published:** 2018-02-26

**Authors:** B. Zhang, W. Ma, Q. Zhu, W. Xu, L. Gao, B. Xu, S. Xu, C. Gao, L. Gao, J. Liu, Y. Cui

**Affiliations:** ^1^ State Key Laboratory of Reproductive Medicine Clinical Center of Reproductive Medicine First Affiliated Hospital Nanjing Medical University Nanjing China; ^2^ Department of Obstetrics and Gynecology Clinical Medical College of Yangzhou University Yangzhou China; ^3^ Department of Gynecology Second Affiliated Hospital Nanjing Medical University Nanjing China

**Keywords:** CYP17a1, Leydig cells, SET protein, StAR, testosterone

## Abstract

Approximately 40% of middle‐aged men exhibit symptoms of late‐onset hypogonadism (LOH). However, the mechanism of androgen deficiency is still currently unclear. As shown in our previous studies, the SET protein is expressed in testicular Leydig cells and ovarian granule cells. This study was designed to investigate the effect of the SET protein on androgen production in Leydig cells. The AdCMV/SET and AdH1siRNA/SET adenoviruses were individually transduced into a cultured mouse Leydig cell line (mLTC‐1) with or without human chorionic gonadotropin (HCG) stimulation in vitro. The primary mouse Leydig cells were used to confirm the main data from mLTC‐1 cells. The SET protein was expressed in the cytoplasm and nucleus of mLTC‐1 cells. Testosterone production was significantly increased in mLTC‐1 cells overexpressing the SET protein compared with the control group (*p *<* *0.05), whereas testosterone production was significantly decreased in the SET knockdown mLTC‐1 cells (*p *<* *0.05). Consistent with the testosterone levels, the expression levels of the steroidogenic acute regulatory (StAR) and cytochrome P450c17α‐hydroxylase (CYP17a1) mRNAs and proteins synchronously changed according to the expression level of the SET protein. Interestingly, the expression of the SET protein was significantly increased in the mLTC‐1 cells stimulated with 0.04 and 0.1 U/mL hCG. In the mLTC‐1 cells transfected with AdH1siRNA/SET and concurrently stimulated with 0.1 U/mL hCG, both testosterone production and StAR expression were significantly lower than in the cells without SET knockdown (*p *<* *0.05). In conclusion, the SET protein participates in regulating testosterone production by increasing the expression of StAR and CYP17a1, and it may be a downstream factor of the classic luteinizing hormone (LH)/luteinizing hormone receptor (LHR) signaling pathway. This study improves our understanding of the intracellular mechanism of testicular steroidogenesis and the pathophysiological mechanism of LOH in the aging male.

## Introduction

Approximately 40% of middle‐aged men exhibit symptoms of late‐onset hypogonadism (LOH), also known as partial androgen deficiency in the aging male (PADAM) (Lunenfeld *et al*., 2005). Many symptoms are related to LOH, including a poor morning erection, erectile dysfunction, low sexual desire, sleep disturbances, and changes in moods, as well as more general symptoms such as insomnia, fatigue, loss of muscle mass, increased fat mass, decreased bone mineral density and osteoporosis, depression, and forgetfulness (Wu *et al*., 2010). LOH has even been shown to be related to substantially higher risks of all‐cause and cardiovascular mortality in aging males (Pye *et al*., 2014). The core mechanism of LOH is an absolute or relative androgen deficiency due to decreases in the number and function of testicular Leydig cells (Lunenfeld *et al*., 2005). However, the mechanism and pathophysiology of LOH are complicated and not completely understood at present.

The main functions of the testis are spermatogenesis and androgen production. Androgens are a class of steroid hormones that includes testosterone (T) and dihydrotestosterone (DHT), and these hormones, together with androgen receptors (ARs), play important roles in promoting male sexual differentiation and pubertal development, the maintenance of secondary sex characteristics, maturation of sexuality, and male fertility (Schlatt & Ehmcke, 2014). T synthesis in testicular Leydig cells requires a variety of key factors and steroidogenic enzymes, such as steroidogenic acute regulatory (StAR) protein, cytochrome P450c17α‐hydroxylase (P450c17, CYP17a1), and 3β‐hydroxysteroid dehydrogenase (HSD3b1). The first rate‐limiting step in androgen synthesis is the facilitation of cholesterol entry into the mitochondria by StAR (Orly & Stocco, 1999). Moreover, the CYP17a1 encoded by the *CYP17a1* gene is a highly active enzyme as both a 17α‐hydroxylase and C17, 20 lyase (Nakajin & Hall, 1981).

The synthesis and secretion of T are classically regulated by the hypothalamus‐pituitary‐gonadal axis (HPG), in which luteinizing hormone (LH) is released from the anterior pituitary gland in pulses upon the stimulation with hypothalamus gonadotropin‐releasing hormone (GnRH). LH induces T production in testicular Leydig cells. Exogenous hCG acts similar to LH through the same LH receptor (LHR) (Saez, 1994; Park *et al*., 2013). In Leydig cells, LH or HCG binds to LHR to activate adenylyl cyclase and convert ATP into cAMP. Then, protein kinase A (PKA) is activated by cAMP, and the activated PKA phosphorylates steroidogenic enzymes or activates transcription factors to promote the expressions of factors and steroidogenic enzymes, such as StAR and CYP17a1. Meanwhile, the cytomembrane Ca^2+^ channel is activated (Chauvigne *et al*., 2012). Both cAMP and Ca^2+^ are secondary messengers in the classical LH signaling pathways. Steroidogenesis is modulated by the complex networks involving endocrine, autocrine and paracrine factors, including insulin‐like growth factor, prolactin, and interleukin 1 (IL‐1) (Saez, 1994). As shown in our previous studies, the SET (for ‘*SE* translocation’) protein is an intracellular factor related to androgen synthesis in ovarian steroidogenesis cells (Gao *et al*., 2013; Xu *et al*., 2013a).

*SET* was originally identified as a translocated gene fused to the *CAN* gene in a patient with acute undifferentiated leukemia (von Lindern *et al*., 1992). The SET protein, also known as template activating factor 1β (TAF‐1β) (Nagata *et al*., 1995), inhibitor of protein phosphatase 2A (I2PP2A) (Li *et al*., 1996), and inhibitor of acetyltransferases (INHAT) (Seo *et al*., 2001), belongs to a family of multitasking proteins that are involved in the cell cycle, cell proliferation, apoptosis, DNA repair, transcription, and epigenetic regulation. The SET protein plays important roles in facilitating cell growth and proliferation via many pathways that promote tumorigenesis and metastasis (von Lindern *et al*., 1992). The SET protein is also expressed at high levels in developing gonads, gonadal steroid hormone‐producing cells (adrenal cells, ovarian theca and granulosa cells and testicular Leydig cells), mature oocytes, and spermatocytes (Xu *et al*., 2013a; Dai *et al*., 2014), suggesting that the SET protein is also involved in regulating steroid hormone synthesis. According to the results from our previous study, the SET protein plays a positive role in regulating ovarian androgen biosynthesis by increasing the transcription of steroidogenic enzymes CYP17a1 and HSD3b1, and the SET‐initiated and PP2A‐mediated pathways lead to the increased lyase activity of CYP17a1 and testosterone biosynthesis (Gao *et al*., 2013; Xu *et al*., 2013b). In addition, the SET protein is expressed in mouse Leydig cells, although its roles in regulating androgen production in the testis are still unclear.

We hypothesize that the SET protein acts as an intracellular regulator that promotes steroidogenesis in testicular Leydig cells. The mouse Leydig cell line mLTC‐1 was used as an in vitro model in which the SET protein was overexpressed or downregulated to test this hypothesis and explore the regulatory effect of the SET protein on androgen production. This study improves our understanding of the intracellular mechanism of testicular steroidogenesis and the pathophysiological mechanism of LOH in the aging male.

## Materials and Methods

### Chemicals and reagents

RPMI‐1640 was purchased from HyClone (Logan, UT, USA), and FBS was purchased from GIBCO BRL (Grand Island, NY, USA). The whole‐cell lysis assay kit was obtained from KeyGEN BioTECH (Nanjing, Jiangsu, China), and the bicinchoninic acid (BCA) protein assay kit and pancreatic enzymes were obtained from Beyotime Biotechnology (Shanghai, China). HCG was obtained from Livzon Pharmaceutical Group (Zhuhai, Guangdong, China). The rabbit polyclonal anti‐SET antibody was obtained from Santa Cruz Biotechnology Inc. (Santa Cruz, CA, USA), the rabbit monoclonal anti‐glyceraldehyde 3‐phosphate dehydrogenase (GAPDH) antibody was obtained from ABCAM (Cambridge Science Park, Cambridge, UK), the rabbit monoclonal anti‐ CYP17a1 antibody was obtained from ABGENT (San Diego, CA, USA), and the rabbit monoclonal anti‐StAR antibody (used as a calibrator) was obtained from Cell Signaling Technology (Denver, CO, USA). Anti‐rabbit horseradish peroxidase (HRP)‐conjugated secondary antibodies were purchased from Jackson Immunoresearch (West Grove, PA, USA). TRIzol was acquired from Gibco (Grand Island, NY, USA). The PrimeScript RT Kit (Perfect Real Time) and SYBR Premix Ex Taq (Perfect Real Time, Takara Bio Inc, Kusatsu, Shiga Prefecture, Japan) were obtained from TaKaRa Biotechnology Co., Ltd. (Dalian, China). Radioimmunoassay (RIA) kits were purchased from the Beijing North Institute of Biological Technology (Beijing, China). The enhanced chemiluminescence (ECL) reagents were obtained from TaKaRa (Shiga, Japan). The mounting medium was obtained from Beyotime (Shanghai, China).

### Construction of the recombinant adenoviruses

The recombinant AdH1siRNA/NS and AdH1siRNA/SET adenoviruses were previously constructed and validated by our laboratory (Gao *et al*., 2013; Xu *et al*., 2013b) using the AdEasy system (He *et al*., 1998). Briefly, the SET cDNA sequence was cloned using reverse transcription polymerase chain reaction (RT‐PCR) and then subcloned into pAdTrack‐CMV. The AdCMV/SET vector was recombined with the backbone pAdEasy‐1 vector in *Escherichia coli* DH5α. The recombined adenovirus AdCMV/SET was generated and amplified in 293T cells, and the order of its elements was the cytomegalovirus promoter (CMV)‐multiple cloning site (MCS)‐3X FLAG tag‐SV40‐EGFP. The titer of the viral stock was determined using the tissue culture transduced dose (TCID50) method. Moreover, the AdCMV adenovirus was generated using an ‘empty’ pAdTrack‐CMV vector as the control. For the endogenous SET knockdown experiments, the recombinant AdH1siRNA/SET adenoviruses carrying siRNAs targeting SET and AdH1siRNA‐Scrambled (control) were constructed as previously described (Shen *et al*., 2003), with certain modifications. The target sequences used for RNA interference were as follows: SET siRNA, 5′‐TCTTCAAAGTCCACCGAAA‐3′ (sense) and SET Scramble, 5′‐TTCTCCGAACGTGTCACGT‐3′. The target sequence was inserted into the pShuttle‐H1 plasmid. The H1‐SiRNA/SET fragment was digested and inserted into the pAdTrack‐CMV plasmid to construct a shuttle plasmid containing the green fluorescent protein (GFP) reporter gene. AdH1siRNA/SET was recombined with the backbone pAdEasy‐1 vector in *Escherichia coli* DH5α. The AdH1siRNA/NS adenovirus was constructed at the same time. The percent transduction efficiencies were monitored by detecting GFP expression under a fluorescence microscope and were approximately 100% in the cultured 293 cell line and mLTC‐1 cells. Adenovirus generation, amplification, and titering were performed using the methods described above.

### MLTC‐1 cell line and the primary culture of mouse Leydig cells

MLTC‐1 cell, a mouse Leydig cell line, was purchased from the Cell Institute of Shanghai (Shanghai, China) and has been used in many previous studies (Sun *et al*., 2012; Gu *et al*., 2015; Klett *et al*., 2016). Testosterone production was stabilized and persistent in the same batch of mLTC‐1 cells; however, differences in T production were observed between batches of cells. T production was assessed before this group of experiments as a reference. All experiments examining T production were repeated more than five times using cells from the same batch. The mLTC‐1 cells were incubated in RPMI‐1640 medium four supplemented with 10% fetal bovine serum, 100 U/mL penicillin and 100 g/mL streptomycin.

The primary mouse Leydig cells were used to confirm the main data from mLTC‐1 cells. Male C57BL/6J mice (4–6 weeks) were purchased from Vital River Laboratory Animal Technology (Beijing, China). Mice were sacrificed by cervical dislocation, and testes were dissected. Testes were washed twice in PBS. The connective tissues and tunica albuginea were then removed, and the pair of testes were incubated in a 50 mL tube with 5 mL 0.25% (w/v) collagenase I (Sigma, St. Louis, MO, USA) at 37 °C for 30 min, rotating at 1000 ***g***. The digestion was stopped by adding 20 mL DMEM‐F12 containing 10% FBS to the mixture. The supernatant was then filtered with 400 mesh stainless screen and centrifuged at 1500 ***g*** for 5 min to pellet the cells. After washing with PBS twice, the cells were finally suspended and in vitro cultured in DMEM‐F12 medium (GIBCO BRL) with 10% FBS (GIBCO) at 37 °C, 5% CO_2_.

### Transfection with the recombinant adenoviruses

The recombinant AdCMV/SET and AdCMV/NS (control for the overexpression of the SET protein) and AdH1siRNA/SET and AdH1siRNA/NS (control for the knockdown expression of the SET protein) adenoviruses were then separately added to the culture media of the mLTC‐1 cells and evenly distributed by gentle shaking. In this study, the primary Leydig cells were also transfected with the AdH1siRNA/SET and AdH1siRNA/NS adenoviruses. After 24 h of transfection, the media was replaced with fresh media. Virus expression was monitored by assessing GFP expression using fluorescence microscopy. Those cells transfected with the recombinant AdH1siRNA/SET or AdH1siRNA/NS adenoviruses were further treated with or without hCG (0.1 U/mL for mLTC‐1 cells, 1.0 U/mL for mLTC‐1 cells) for 4 h before harvest to explore the overlapping effects of both exogenous LH and the endogenous SET protein. The cells were cultured for 72 h after transfection and the hCG treatment, harvested for qRT‐PCR, and stored at −80 °C for a subsequent Western blot analysis. The culture media was collected and stored at −80 °C for the hormone assay.

### Immunofluorescence assay and confocal laser scanning microscopy

The mLTC‐1 cells were fixed with 4% paraformaldehyde (pH 7.0) for 30–60 min at room temperature min. After four washes with PBS, the cells were permeabilized with PBS containing 0.4% Triton X‐100 for 5 min at room temperature. The cells were washed three times in PBS and then incubated with 5% bovine serum albumin (BSA) for 2 h at 37 °C. The cells were incubated with a rabbit polyclonal anti‐SET (1 : 50) antibody overnight at 4 °C, and cells incubated without the primary antibodies were used as the negative control. Subsequently, all cells were washed three times with PBS and incubated with a goat anti‐rabbit IgG‐fluorescein isothiocyanate antibody (IgG‐FITC, A‐11012, 1 : 500) from Invitrogen (Carlsbad, CA, USA) for 1 h at 37 °C in the dark. All the cells were washed three times with PBS, and all the nuclei were stained with 4′, 6‐diamidino‐2‐phenylindole (DAPI) for 2 min. Finally, all cells were placed in mounting medium on slides and covered with ethanol‐primed coverslips. The cells were stored in the dark before confocal scanning. Immunostained cells were observed with a laser scanning confocal microscope (LSM710; Carl Zeiss, Oberkochen, Germany) under a 20× oil objective. The cells were scanned in 15 optical sections from the top to the bottom of the cells. FITC and DAPI were detected at wavelengths of 561 and 405 nm, respectively. The parameters were set at constant values for all measurements. Ultimately, the images captured from the different color channels were merged and further processed using zeiss lsm image browser software (Carl Zeiss, Oberkochen, Southwest of Germany, Germany).

### RNA extraction and qRT‐PCR

RNA was extracted from the mLTC‐1 cells or primary mouse Leydig cells using TRIzol reagent (Invitrogen), and total RNA was reverse transcribed using a PrimeScript Reverse Transcription Kit (Perfect Real Time) according to the manufacturer's instructions. Amplification reactions were conducted using the SYBR Premix Ex Taq (Perfect Real Time) on an ABI PRISM 7300 system. The primers for the mouse *SET*,* StAR*,* CYP17a1, CYP11a1,* and *HSD3b1* genes were as follows: *SET*‐PF: GTCCACCGAAATCAAATGGAAATC, *SET*‐PR: GCACCTGCATCAGAATGGTCA; *StAR*‐PF: CCACCTGCATGGTGCTTCA, *StAR*‐PR: TTGGCGAACTCTATCTGGGTCTG; *CYP17a1*‐PF: TCTGGGCACTGCATCACG, *CYP17a1*‐PR: GCTCCGAAGGGCAAATAACT; *CYP11a1*‐PF: AGTTCAGATGCCTGGAAGAAAGA, *CYP11a1‐PR*: ACTCAAAGGAAAAGCGGAATAGG; *HSD3b1*‐PF: GTTTGTGGGCCAGAGGATCA, *HSD3b1*‐PR: GGTCTTTGTCTGCAGCTTGGA. The *GAPDH*, a housekeeping gene, was employed as an internal reference gene to normalize the loading of the cDNA templates. The primers for the mouse *GAPDH* gene were *GAPDH*‐PF: AGGTCGGTGAACGGATTTG and *GADPH*‐PR: GGGGTCGTTGATGGCAACA. A melting curve analysis was performed to confirm the specificity of the qRT‐PCR products. Each sample was assayed in duplicate, and the fold change in the expression of each gene of interest was analyzed using the 2^−ΔΔCt^ method and is presented as the fold change normalized by *GAPDH* (Livak & Schmittgen, 2001).

### Hormone assays

The T levels in the conditioned media were assessed using radioimmunoassay (RIA) kits according to the manufacturer's protocol. The RIA kits were sensitive (<0.02 ng/mL) and reproducible (total coefficient of variation [CV], <10%), with measurements ranging from 0.1 to 20 ng/mL.

### Western blot analysis

Total proteins were extracted from the cultured mLTC‐1 cells or primary mouse Leydig cells using radioimmunoprecipitation (RIPA) lysis buffer according to the Bradford method. Protein concentrations were determined using BCA kits. Fifty micrograms of total protein was separated using SDS‐PAGE and then transferred to polyvinylidene difluoride (PVDF) membranes. The membranes were incubated with specific anti‐SET (1 : 1000), anti‐GAPDH (1 : 1000), anti‐CYP17a1 (1 : 200), and anti‐StAR (1 : 1000) antibodies. The GAPDH, a housekeeping protein, was employed as an internal reference to normalize the loading of total protein. After the membranes were incubated with secondary antibodies, an ECL kit was used to detect the signals. The bands were quantified using the analytical software provided with the imaging system.

### Statistical analysis

Data are expressed as the mean ± standard errors of the means (SEM) from five to six independent experiments. The one‐way analysis of variance (anova) was used to evaluate generally the difference between groups, and the paired sample *t*‐test was used for statistical comparisons between two groups. Differences were considered significant at *p *<* *0.05 and *p *<* *0.01.

## Results

### Expression of the SET protein in the cultured mLTC‐1 cells

**Figure 1:**
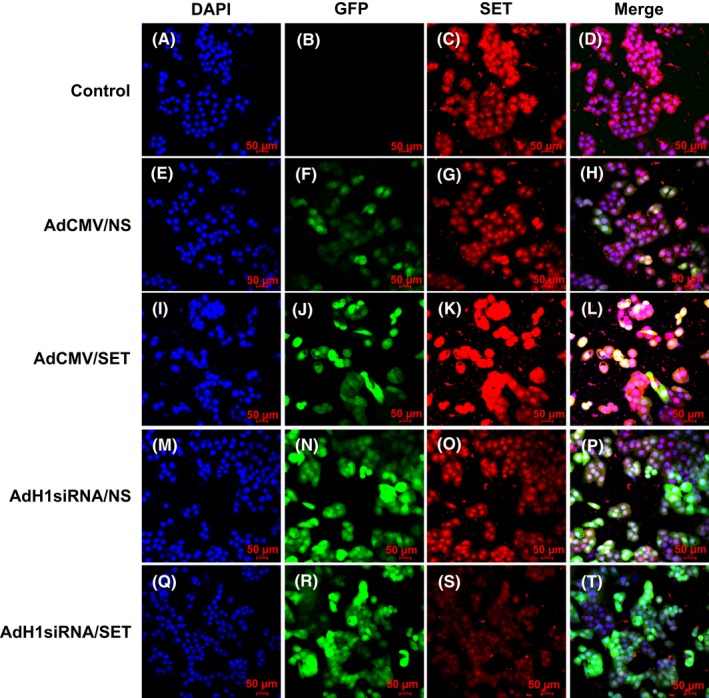
Expression of the SET protein in the cultured mLTC‐1 cells. The mLTC‐1 cells in the control group were not transfected with adenovirus, and the other groups were treated with AdCMV/NS, AdCMV/SET, AdH1siRNA/NS, or AdH1siRNA/SET. Nuclei were stained with DAPI (A, E, I, M, and Q). SET‐positive staining was shown in red. (C, G, K, O, and S) The expression of the GFP protein from the transfected adenovirus was shown in green (B, F, J, N and R). Immunofluorescence staining for the SET protein and nuclear DAPI signal and GFP protein were merged (D, H, L, and T). SET‐positive staining was observed in mLTC‐1 cells. The SET protein was expressed in both the cytoplasm and nucleus of mLTC‐1 cells, and the intensity of the nuclear staining was greater than the cytoplasmic staining. Bar = 50 μm.

The subcellular location of SET immunofluorescence staining was observed using a laser scanning confocal microscope to confirm that the SET protein was expressed in the cultured mLTC‐1 cells (Fig. 1). The SET protein was observed in both the cytoplasm and nucleus of the mLTC‐1 cells; however, the intensity of the nuclear staining was significantly greater than the cytoplasmic staining. The location and expression of the SET protein in cultured mLTC‐1 cells were similar in the cells transfected with AdH1siRNA/NS and with AdCMV/NS (Fig. 1C,G,O). The intensity of the SET staining in the cells transfected with AdCMV/SET was greater than that of the control group transfected with AdCMV/NS (Fig. 1K,G) and vice versa (Fig. 1S,O).

### Effect of the SET protein on T production in the mLTC‐1 cells

**Figure 2:**
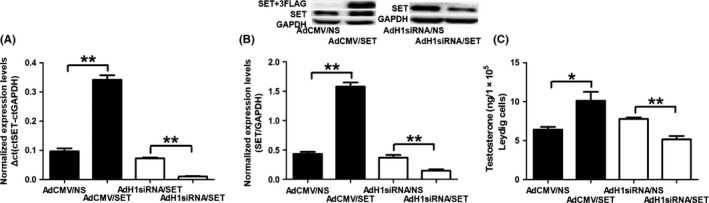
Effect of the SET protein on T production in mLTC‐1 cells. (A) Expression of the SET mRNA in the mLTC‐1 cells transfected with AdCMV/SET and AdH1siRNA/SET adenoviruses. (B) Expression of the SET protein in the mLTC‐1 cells transfected with AdCMV/SET and AdH1siRNA/SET adenoviruses. The order of the elements in the recombined AdCMV/SET adenovirus was CMV‐MCS‐3FLAG‐SV40‐EGFP. Thus, the total expression level of the SET protein in the mLTC‐1 cells transfected with AdCMV/SET adenoviruses was the SET protein plus the SET‐3FLAG protein. (C) T production in the mLTC‐1 cells transfected with AdCMV/SET and AdH1siRNA/SET adenoviruses. *p <* *0.05, **p <* *0.01.

The cultured mLTC‐1 cells were transfected with AdCMV/SET adenoviruses to induce the overexpression of SET protein or with AdH1siRNA/SET adenoviruses to knockdown the expression of SET protein. AdCMV/NS or AdH1siRNA/NS adenoviruses were used as the controls. The efficacy was validated at both the mRNA and protein levels (Fig. 2A,B). The expression levels of the SET mRNA and protein were significantly increased by over 3‐fold in the mLTC‐1 cells transfected with AdCMV/SET adenoviruses (*p *<* *0.01), whereas the levels were decreased by approximately 80–90% in the mLTC‐1 cells transfected with AdH1siRNA/SET adenoviruses (*p *<* *0.01). T level was significantly increased by 58% in the conditioned media of the mLTC‐1 cells transfected with AdCMV/SET adenoviruses compared with the control group (Fig. 2C, *p* < 0.05), whereas this value was significantly decreased by 33% in the AdH1siRNA/SET‐transfected group (Fig. 2C, *p* < 0.01). Based on these results, the SET protein stimulates T production in Leydig cells.

### Effect of the SET protein on key regulators of steroidogenesis in mLTC‐1 cells

**Figure 3:**
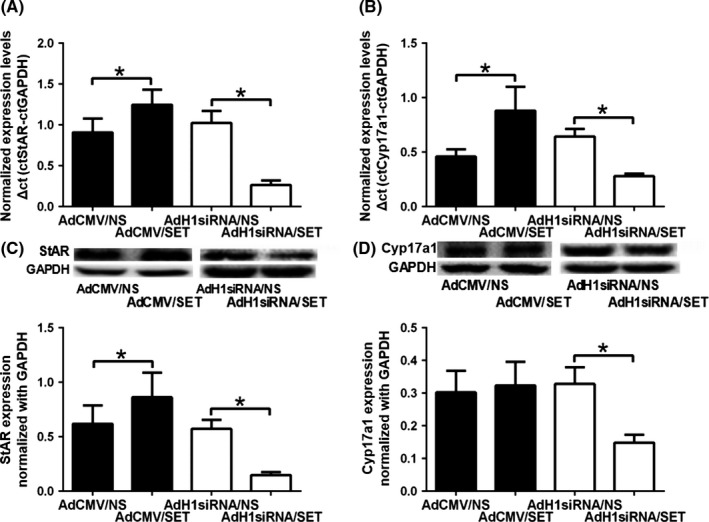
Effect of the SET protein on key regulators of steroidogenesis in mLTC‐1 cells. The cultured mLTC‐1 cells were transfected with AdCMV/NS AdCMV/SET, AdH1siRNA/NS, or AdH1siRNA/SET adenoviruses. (A) Expression of the *StAR* mRNA in the mLTC‐1 cells transfected with AdCMV/SET or AdH1siRNA/SET adenoviruses. (B) Expression of the *CYP17a1* mRNA in the mLTC‐1 cells transfected with AdCMV/SET or AdH1siRNA/SET adenoviruses. (C) Expression of the StAR protein in the mLTC‐1 cells transfected as described above. (D) Expression of the CYP17a1 protein in the mLTC‐1 cells transfected as described above. Expressions of target genes and proteins were normalized by the GAPDH. *p <* *0.05.

The expressions of StAR and CYP17a1 were measured in the mLTC‐1 cells transfected with AdCMV/SET or AdH1siRNA/SET adenoviruses to explore whether the SET protein upregulated androgen production by increasing the expressions of enzymes involved in steroidogenesis (Fig. 3). In the mLTC‐1 cells transfected with AdCMV/SET adenoviruses, the expressions of both the StAR mRNA and protein were increased by approximately 38% and the expression of the CYP17a1 mRNA as significantly increased by 91% compared with those in the control group (*p *<* *0.05). The expression of the CYP17a1 protein only exhibited a slight increase in the cells transfected with the AdCMV/SET adenovirus (*p *>* *0.05). In the mLTC‐1 cells transfected with AdH1siRNA/SET adenoviruses, the expressions of the StAR and CYP17a1 mRNAs and proteins were significantly decreased by approximately 75–80% and 55–60%, respectively, compared with the control (*p *<* *0.05). Thus, the SET protein promotes androgen production in Leydig cells by upregulating the expressions of key factors and enzymes involved in steroidogenesis, such as StAR and CYP17a1.

### Effect of the SET protein in the primary mouse Leydig cells

**Figure 4:**
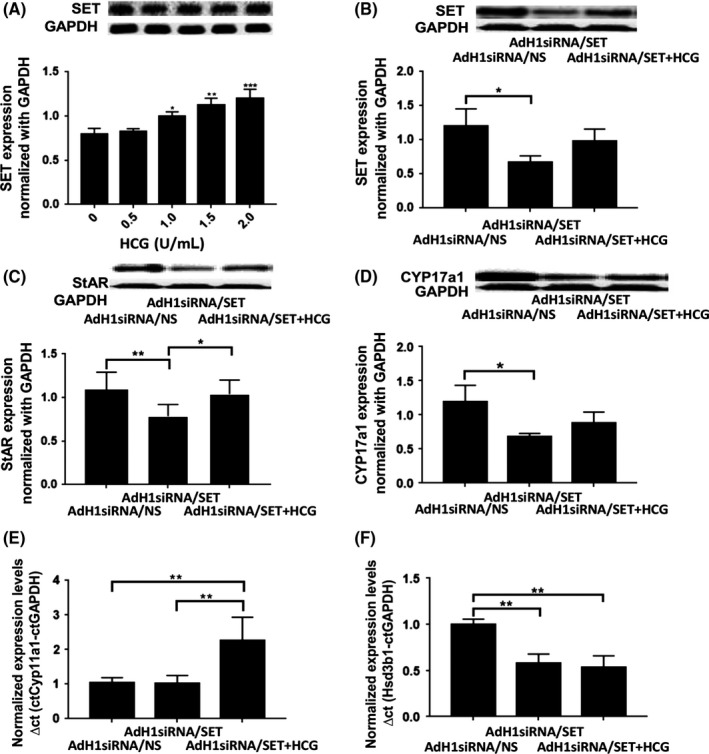
Effect of the SET protein in the primary mouse Leydig cells. To confirm the data from mLTC‐1 cells, the mouse Leydig cells were isolated and cultured in vitro. The primary Leydig cells were infected with AdH1siRNA/SET adenoviruse to downregulate the expression of SET protein. (A) The expression of SET protein in the primary Leydig cells was increased by hCG treatment in a dose‐dependent manner (0 to 2.0 IU/mL). (B) The expression of SET protein was significantly downregulated (50%) by AdH1siRNA/SET adenoviruse and increased in a modicum by hCG treatment. (C) The expression of StAR protein in the primary Leydig cells transfected with AdH1siRNA/SET adenoviruse. (D) The expression of CYP17a1 protein in the primary Leydig cells transfected with AdH1siRNA/SET adenoviruse. (E&F) The expressions of CYP11a1 and HSD3b1 mRNAs in the primary mouse Leydig cells were tested. Expressions of target genes and proteins were normalized by the GAPDH. *p <* *0.05, **p <* *0.01.

To confirm the data from mLTC‐1 cells, the mouse Leydig cells were isolated and cultured in vitro (Fig. 4). The primary Leydig cells were infected with AdH1siRNA/SET adenoviruse to downregulate the expression of SET protein. The expression of SET protein in the primary Leydig cells was increased by hCG treatment in a dose‐dependent manner (0 to 2.0 IU/mL) (Fig. 4A, *p* < 0.05 or *p* < 0.01). The expression of SET protein was significantly downregulated (50%) by AdH1siRNA/SET adenoviruse (Fig. 4B, *p* < 0.05) while this expression was increased in a modicum by the additional hCG treatment (but *p* > 0.05). Correspondingly, the expressions of StAR and CYP17a1 proteins in the primary Leydig cells were significantly decreased by the downregulation of SET protein (Fig. 4C, *p* < 0.01; Fig. 4D, *p* < 0.05). With the additional hCG treatment, the expression of StAR protein was significantly increased in a qualified sense (Fig. 4C, *p* < 0.05) while the expression of CYP17a1 was also increased (Fig. 4D, although *p* > 0.05).

Additionally, the expressions of CYP11a1 and Hsd3b1 in the primary mouse Leydig cells were tested (Fig. 4E,F). The expressions of CYP11a1 and HSD3b1 mRNAs were tested using qPCR. The expression of HSD3b1 in the cells with SET downregulation was significantly lower than that in the control (*p* < 0.01) although the change in Cyp11a1 was not significant (*p* > 0.05). Interestingly, the expression of CYP11a1 in the primary Leydig cells with SET downregulation was significantly increased by the additional hCG treatment (*p* < 0.01) while the expression of HSD3b1 was not rescued by the additional hCG treatment (*p* > 0.05).

### Relationship between the SET protein and the LH/LHR signaling pathways

**Figure 5:**
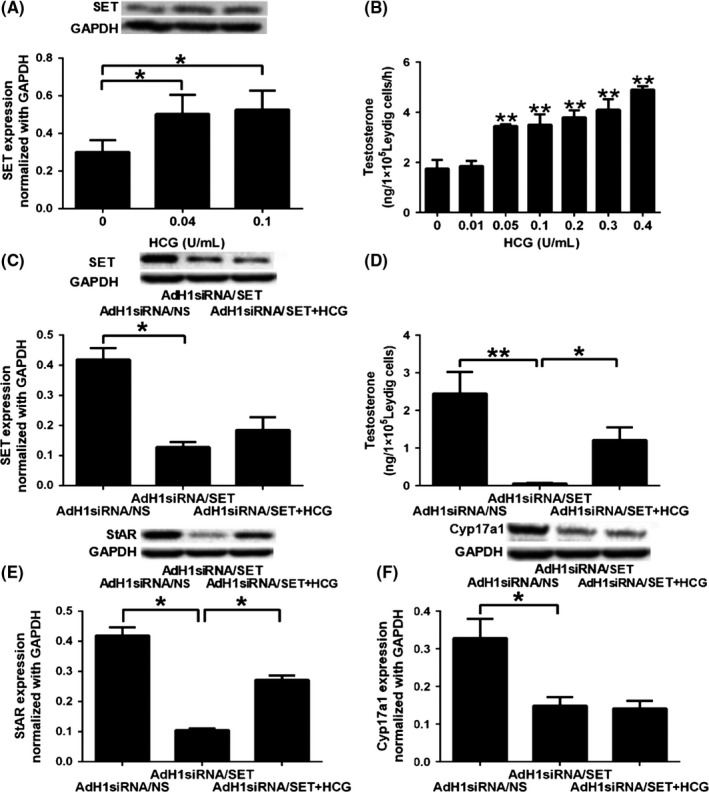
Relationship between the SET protein and the classical LH pathways. (A) Expression of the SET protein in the mLTC‐1 cells treated with 0, 0.04, or 0.1 U/mL hCG for 4 h. (B) T production in the mLTC‐1 cells treated with increasing concentrations of hCG (0.01–0.4 U/mL). (C) Expression of the SET protein in the mLTC‐1 cells transfected with AdH1siRNA/SET adenoviruses for 72 h and then treated with 0.1 U/mL hCG for 4 h. (D) T production in the mLTC‐1 cells treated as described in (C). (E) Expression of StAR protein in the SET knockdown mLTC‐1 cells treated with 0.1 U/mL hCG. (F) Expression of CYP17a1 protein in the mLTC‐1 cells treated as described in (E). *p <* *0.05, **p <* *0.01.

The mLTC‐1 cells were treated with hCG ranging from 0 to 0.4 U/mL to determine the optimal concentration of hCG (Fig. 5A,B). Treatment with 0.04 or 0.1 U/mL hCG for 4 h significantly increased the expression of the SET protein by 67% or 74%, respectively (Fig. 5A, *p* < 0.05). T production was noticeably increased by up to 2‐fold in the mLTC‐1 cells treated with 0.05 to 0.4 U/mL hCG in a dose‐dependent manner (Fig. 5B, *p* < 0.01). Therefore, the treatment concentration of hCG was 0.1 U/mL in the following experiments. The expression of the SET protein was significantly inhibited by 70–80% in the mLTC‐1 cells transfected with AdH1siRNA/SET adenoviruses (Fig. 5C, *p* < 0.05), and T production was also significantly inhibited by more than 90% (Fig. 5D, *p* < 0.01). When the SET knockdown cells were simultaneously treated with 0.1 U/mL hCG, the expression of the SET protein increased (compared with the SET knockdown cells without hCG stimulation, although *p *>* *0.05), and T production was significantly increased by over 22‐fold (*p *<* *0.05). Moreover, T production was significantly reduced in the SET knockdown cells that were simultaneously treated with hCG when compared with the AdH1siRNA/NS group (Fig. 5D, *p* < 0.05) or the cells stimulated with 0.1 U/mL hCG (Fig. 5B, *p* < 0.05). In the SET knockdown mLTC‐1 cells, the expressions of the *StAR* and *CYP17a1* mRNAs were significantly inhibited by 80% and 55%, respectively (Fig. 5E,F, *p* < 0.05). The expression of the StAR protein was rescued by approximately 40% by the hCG treatment (Fig. 5E, *p* < 0.05), but the expression of the CYP17a1 protein was not rescued (Fig. 5F, *p* > 0.05).

## Discussion

The core mechanism of LOH in the aging male is the absolute or relative androgen deficiency due to the decrease in the number and function of testicular Leydig cells (Lunenfeld *et al*., 2005). The LH/LHR pathway is the classical pathway involved in regulating T production in Leydig cells. Many intratesticular factors and intracellular factors participate in regulating steroidogenesis (Saez, 1994). In a previous study, we observed the expression of SET protein in the testes of mice at different developmental stages (Dai *et al*., 2014). The expression of SET protein was found in Leydig cells. The expression level of SET protein in the aging group was significantly lower than that in the adult group (*p* < 0.05), while the mean of *SET* mRNA expression was also lower in the aging group (although *p* > 0.05). The expression of SET protein in Leydig cells suggests a possible role in steroidogenesis as an intracellular factor of testicular Leydig cells (Dai *et al*., 2014). In this study, we found that the SET protein was a potential downstream factor of LH/LHR signaling with a stimulatory effect on T production in Leydig cells by upregulating the expressions of key factors and enzymes involved in steroidogenesis, such as StAR, CYP17a1, CYP11a1, and HSD3b1. These results definitely help us understand the mechanism of androgen deficiency in aging males with LOH.

The SET protein is a 39 kDa phosphoprotein that is widely expressed in various tissues, particularly in steroidogenic cells within the central nervous system, adrenal glands, and gonads (Nagata *et al*., 1998; Compagnone *et al*., 2000). The expression of the SET protein in human NT2 neuronal precursor cells suggests that it has a role in neuro‐steroidogenesis (Compagnone *et al*., 2000). In rat ovaries, the SET protein is expressed in theca cells and oocytes (Zhang *et al*., 2001). As shown in our previous study, the SET protein is mainly expressed in human theca cells and overexpressed in polycystic ovarian tissues, suggesting that the SET protein regulates ovarian androgen biosynthesis and participates in the pathophysiology of hyperandrogenism in polycystic ovary syndrome (PCOS) (Diao *et al*., 2004; Gao *et al*., 2013; Xu *et al*., 2013a,b). Moreover, the SET protein is also expressed in the cytoplasm and nucleus of mouse testicular Leydig cells (Dai *et al*., 2014). In this study, we found that the SET protein was expressed in both the nucleus and cytoplasm of mLTC‐1 cells and that the intensity of the nuclear signal was greater than the cytoplasmic signal. Using the cultured mLTC‐1 cells and the primary mouse Leydig cells with overexpression or knockdown of the SET protein, we confirmed that the SET protein stimulated T production. In vivo studies using loss‐of‐function of the SET protein or the gene knockout models will further confirm this in vitro function of the SET protein, although we have not currently developed those models.

**Figure 6:**
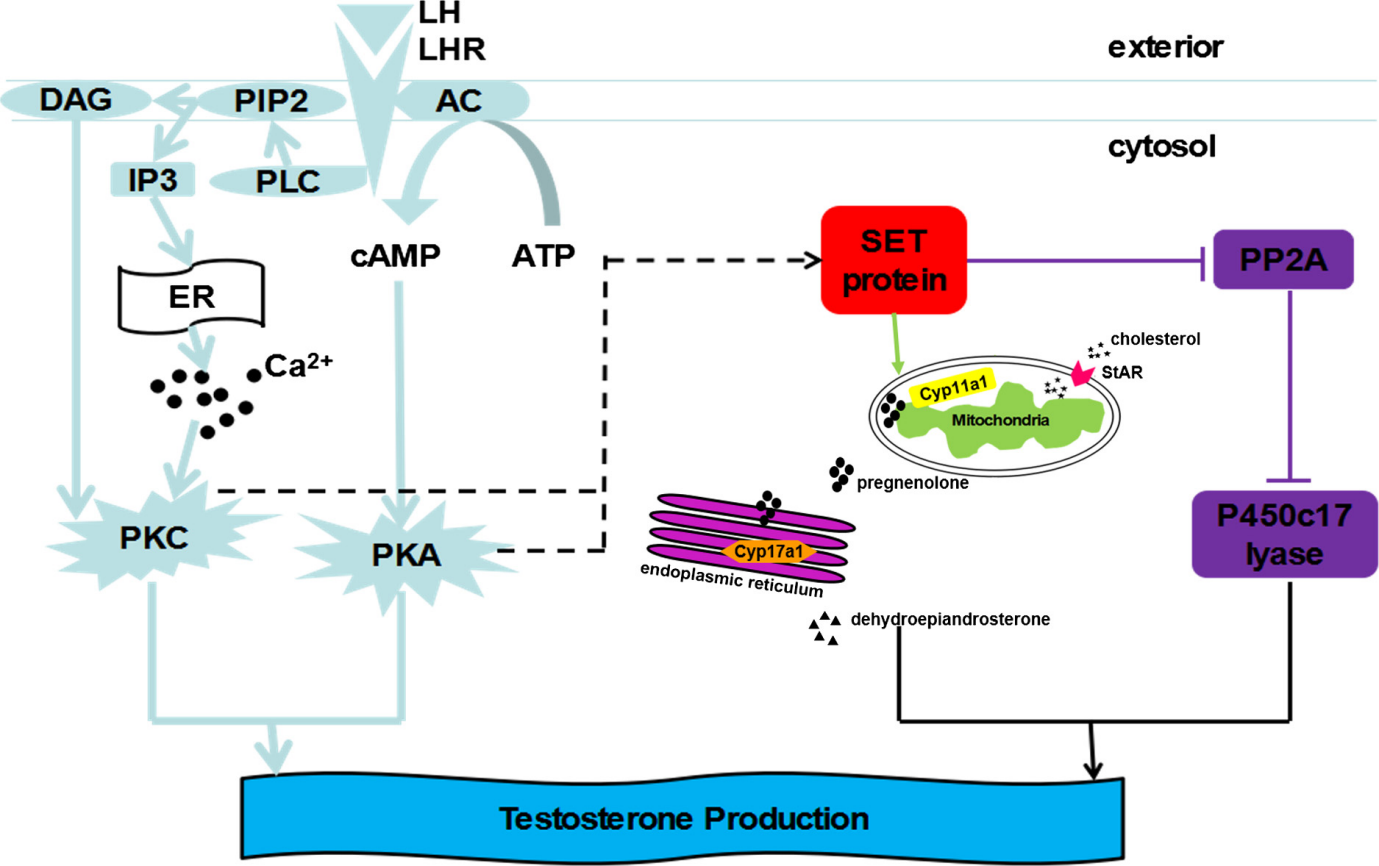
SET protein participates in regulating T production in testicular Leydig cells. The SET protein may act as a downstream factor of two main LH/LHR pathways. The SET protein then upregulates the expression of StAR and key enzymes involved in steroidogenesis, such as CYP17a1, in addition to inhibiting PP2A. The positive effect of the SET protein in Leydig cells is to promote T production. LH: luteinizing hormone; LHR: LH receptor; AC: adenylate cyclase; PKA: protein kinase A; PLC: phospholipase C; PIP2: phosphatidyl inositol 4,5‐bisphosphate; DAG: diacyl glycerol; IP3: inositol triphosphate; PKC: protein kinase C; PP2A: protein phosphatase 2A; ER: endoplasmic reticulum.

The SET protein has been identified as a potent inhibitor of protein phosphatase 2A (PP2A). According to the study by Pandey *et al*., the SET protein facilitates androgen biosynthesis by inhibiting PP2A and fostering the 17, 20 lyase activity of CYP17a1 in adrenals (Pandey *et al*., 2003), because the phosphorylation of CYP17a1 on serine and threonine residues increases 17, 20 lyase activity through unidentified mechanisms, which may involve increasing its affinity for *b*5 and/or P450 oxidoreductase (Zhang *et al*., 1995). As shown in our previous studies, the SET protein plays a positive role in regulating human ovarian androgen biosynthesis by increasing the transcription of the steroidogenic enzymes CYP17a1 and HSD3b1, and the SET‐initiated and PP2A‐mediated pathways lead to the increased lyase activity of CYP17a1 and T biosynthesis, which may contribute to hyperandrogenism in PCOS (Gao *et al*., 2013; Xu *et al*., 2013a,b). These results also advance our knowledge of the function of the SET protein in the regulation of testicular steroidogenesis (Fig. 6). Based on recent evidence, the SET protein is required to regulate both the promoter activity of *CYP17* and the biological activity of CYP17a1 (Zhang *et al*., 2001). In human NT2 neuronal precursor cells and Leydig cells, the SET protein binds to the DNA at a site located at ‐418/‐399 of the rat *CYP17a1* promoter and activates its basal transcription from a rat *CYP17a1* luciferase reporter plasmid (Zhang & Mellon, 1997; Compagnone *et al*., 2000). In the testis of the adult rat, CYP17a1 is only expressed in Leydig cells; the SET protein is also expressed in Leydig cells. Based on the present study in mLTC‐1 and mouse primary cells, the SET protein has a positive effect on T production by upregulating CYP17a1 expression.

In the primary mouse Leydig cells, we found that the expression of *HSD3b1* was significantly regulated by the SET protein while *CYP11a1* was not (Fig. 4). Interestingly, the expression of *CYP11a1* in the primary Leydig cells with the downregulation of SET protein was significantly increased by the additional hCG treatment while the expression of *HSD3b1* was not rescued by the additional hCG treatment. These results of CYP11a1 and HSD3b1 in the primary Leydig cells were in accord with our previous results from mouse ovarian tissues and follicles (Gao *et al*., 2013; Xu *et al*., 2013a,b). CYP11A1 protein resided in the mitochondria catalyzes the first step of steroidogenesis where cholesterol is converted to pregnenolone. HCG is one of regulator of CYP11a1. We concluded that the SET protein participates in the regulation of testosterone production mainly by CYP17a1 and HSD3b1 and that the CYP11a1 could not be the target of SET protein. Translocation of cholesterol from the outer mitochondrial membrane to the relatively sterol‐poor inner membrane is a critical step in steroidogenesis, which is specifically controlled by StAR (Orly & Stocco, 1999). In this study, the SET protein also increased the expressions of the *StAR* mRNA and StAR protein in mLTC‐1 cells and the primary mouse Leydig cells. The SET protein belongs to a family of multitasking proteins that are involved in the SET‐induced regulation of genomic transcription and epigenetics. Our future studies will determine the underlying mechanism by which the SET protein regulates StAR and CYP17a1 expression.

Steroidogenesis is regulated by the classical LH/LHR‐cAMP‐PKA pathway and the phosphatidyl inositol 3,4,5‐triphosphate (PIP3)‐Ca^2+^‐PKC pathway (Fig. 6). LH binds to LHR, a G protein‐coupled receptor located on the membrane of Leydig cells, and activates adenylate cyclase, which converts ATP into cAMP. Then, cAMP activates PKA (Chauvigne *et al*., 2012). In addition, LH/LHR induces the release of Ca^2+^ from the endoplasmic reticulum (ER) to activate PKC (Manna *et al*., 2011). PKA and PKC activate downstream transcription factors to upregulate the expressions of steroidogenic enzymes or directly activate the associated synthetases (Manna *et al*., 2011; Chauvigne *et al*., 2012). In addition, many other pathways such as PKA‐extracellular‐regulated kinase (ERK), Ras‐Raf‐MEK‐ERK, and PI3K‐AKT participate in the downstream signaling of LH/LHR (Saez, 1994). We did not know how the expression of *SET* gene was positively regulated by the LH/LHR‐related PKC and PKA pathways. In the present study, we found that the expression of SET protein in the mLTC‐1 cells and the primary mouse Leydig cells was regulated by hCG stimulation. However, the change in SET protein translocation in response to hCG stimulation was not quantified in this study. The nuclear import of SET protein relies on GTPases and is mediated by an importin alpha (Impα)/Impβ‐dependent pathway. The nuclear localization signal (NLS) in the SET protein, ^168^KRSSQTQNKASRKR^181^, interacts with Impα3, which recruits Impβ to form a ternary complex, resulting in the efficient transport of SET protein into the nucleus (Qu *et al*., 2007). Ser9 is nested in the center of the ^6^AKVSKK^11^ sequence in the SET protein, which is consistent with other classical NLS sequences such as KKXXKX or XKXXKK. Casein kinase II (CKII)‐mediated phosphorylation of Ser9 in the SET protein prohibits the formation of the importin‐SET complex, which induces the cytoplasmic retention of SET protein (Yu *et al*., 2013). Using the SET knockdown mLTC‐1 cell model, we showed that the action of hCG was partially reduced by SET knockdown, and hCG partially rescued the effect SET knockdown on the StAR and CYP17a1 expressions and androgen production. Although additional studies are required to identify the pathways associated with the SET protein, we hypothesize that the SET protein may act as a downstream factor of the LH/LHR signaling pathways (Fig. 6).

## Conclusions

In conclusion, the SET protein is expressed in the cytoplasm and nucleus of Leydig cells and positively regulates T production by upregulating StAR and CYP17a1 expression. The potential mechanism is that the SET protein acts as a downstream factor of the LH/LHR signaling pathways. These findings advance our knowledge of the intracellular mechanism by which the SET protein promotes testicular steroidogenesis, which also helps us understand the pathophysiological mechanisms of male primary hypogonadism and androgen deficiency in the aging male with LOH.

## Funding

The study was supported by grants from the National Natural Science Foundation of China (81370754, 81170559, 81401173, and 31301182) and the Jiangsu Province Special Program of Medical Science (BL2012009 and XK02200901‐NG09).

## Disclosure Information

None of the authors have anything to declare.

## Authors’ Contributions

Zhang B performed the main experiments and wrote the first draft of the manuscript. Ma W finished the experiments of the primary mouse Leydig cells. Zhu Q, Xu W, and Xu S, who are graduate students of Cui Y and Liu J, assisted Zhang in performing the experiments. Gao L and Xu B developed the AdCMV/SET and AdH1siRNA/SET vectors. Gao C and Gao L assisted with the confocal microscopy and RIA. Cui Y designed the study and revised the manuscript. All authors read and approved the final manuscript. We thank He Jing, a postdoctor in Dr Cui's laboratory, for her help in the primary mouse Leydig cells.
